# Numerical Optimization of Refractive Index Sensors Based on Diffraction Gratings with High Aspect Ratio in Terahertz Range

**DOI:** 10.3390/s22010172

**Published:** 2021-12-28

**Authors:** Oleg Kameshkov, Vasily Gerasimov, Boris Knyazev

**Affiliations:** 1 Budker Institute of Nuclear Physics SB RAS, 630090 Novosibirsk, Russia; o.kameshkov@g.nsu.ru (O.K.); ba_knyazev@phys.nsu.ru (B.K.); 2 Department of Physics, Novosibirsk State University, 630090 Novosibirsk, Russia

**Keywords:** terahertz range, linear diffraction gratings, surface plasmon resonance, refractive index sensing

## Abstract

Terahertz surface plasmon resonance (SPR) sensors have been regarded as a promising technology in biomedicine due to their real-time, label-free, and ultrasensitive monitoring features. Different authors have suggested a lot of SPR sensors, including those based on 2D and 3D metamaterials, subwavelength gratings, graphene, and graphene nanotube, as well as others. However, one of the traditional approaches to realize high sensitivity SPR sensors based on metal diffraction gratings has been studied poorly in the terahertz frequency range. In this article, a linear metal rectangular diffraction grating with high aspect ratio is studied. The influence of the grating structure parameters on the sensor sensitivity is simulated. Effects arising from different ratios of depth and width were discovered and explained. The results show that the sensitivity can be increased to 2.26 THz/RIU when the refractive index range of the gas to measure is between 1 and 1.002 with the resolution $5\times 10^{-5}\;\mathrm{RIU}$.

## 1. Introduction

Fast and ultrasensitive surface plasmon resonance (SPR) sensors are a well-proven technology of refractive index and spectra substance measurement in the optical frequency range [1]. It is based on the electron surface wave excitation in the Kretschman scheme on thin metallic films. A surface plasmon can be considered as an evanescent wave propagating along the metal–dielectric interface. The electric field decays normally to the interface, providing a high sensitivity of the method to changes on the surface. The tiny spatial dimension (less than the wavelength) of surface plasmon enables characterization of objects of submicron size.

Terahertz (THz) radiation is the part of electromagnetic radiation between the microwave and infrared regions, which lies in the frequency range of 0.3–3 THz. Terahertz waves are non-ionizing, able to pass through many dry dielectrics, and are strongly absorbed in polar solutions. Owing to the relatively large dimension of terahertz waves, they can be used for study of changes in super micron-sized objects. Terahertz radiation also tends to be very sensitive to the rotation vibration of molecules and thus makes it possible to obtain information that is not available with other analytical and imaging techniques. All this makes terahertz spectroscopy a promising tool for biological research [2,3,4].

High narrow peaks in the spectra and high concentration of the electromagnetic field are required for detection of low concentrations of the substance and tiny changes in the dielectric medium. The use of SPR in the terahertz frequency range on thin metal films is limited due to the high values of the optical constants of metals [5,6]. To overcome this problem, different authors suggest their solutions based on 2D and 3D metamaterials, subwavelength gratings, graphene, and graphene nanotube, as well as others [7,8,9]. For example, metagratings, which can act both as couplers and sensors, attract the increased attention of researchers [10,11,12]. Xiangjun Li et al. have used one of these metagratings and showed that their device achieved a high sensitivity of 335 GHz/RIU and a detection limit less than 0.0001 RIU with a frequency resolution of 33.5 MHz. [12]. Another type of the sensor design based on subwavelength gratings and Otto prism coupler was proposed by Ng et al. [13]. Such sensors were developed in the works of other authors [13,14,15,16,17,18,19,20], who obtained the maximum sensitivity to be as high as 3.57 THz/RIU. The corresponding figure of merit (FOM) is 3966 [20]. However, one of the traditional approaches to realize high sensitivity SPR sensors based on metal diffraction gratings has been studied poorly in the terahertz frequency range.

In 2004, O’Hara et al. was the first to experimentally investigate the excitation of terahertz surface plasmon polariton (SPP) wave with a metal grating and TDS spectroscopy [21]. They determined that the coupling process was efficient, but the lifetime of SPP was very short. This is because the coupling and decoupling processes happened at the same time. Researchers suggested that another lattice profile, dielectric coating, or some other change in the system configuration, would result in a coupling/decoupling compromise. In 2008, Nazarov et al. published an article in which they optimized the excitation of terahertz SPP on structured metallic surfaces by varying grating parameters [22]. They analyzed the governing process of the coupling phenomenon and propagation of SPP over flat and corrugated structures. Other researchers investigated numerically and experimentally diffraction gratings with a trapezoidal profile [23]. They showed how to use multi-section groove gratings for efficient excitation of broadband THz SPPs. In 2011, Nazarov and Coutaz presented a review on SPRs and SPPs on metal diffraction linear grating applications and explorations, but there was no mention of sensing applications [24]. Later, Spevak et al. investigated the features of SPR excitation on small semiconductor diffraction gratings [25]. In 2020, Sathukarn et al. used the rigorous coupled-wave analysis (RCWA) method to design terahertz SPR diffraction linear rectangular gratings providing as a refractive index sensor [26]. They parametrically analyzed the gratings and found optimal parameters for surface plasmon resonance. They showed that their sensor provided a sensitivity of 500 GHz/RIU and a detected resolution of 0.01 RIU, working in the frequency interrogation.

In this paper, we investigate a one-dimensional periodic metal rectangular diffraction grating with high aspect ratio for terahertz SPR sensing. First, we introduce the configuration of the proposed terahertz SPR sensor and its simulation analogue. Next, we analyze the influence of the groove width and depth on the resonance and mode excitation of the diffraction grating. Finally, terahertz SPR sensors with different grating configurations are examined via characterization of analytes with different refractive indices for gas sensing.

## 2. SPR Sensing System—Coupling Mechanism

### 2.1. Coupling of Surface Waves to a Cavity Mode 

**Figure 1:**
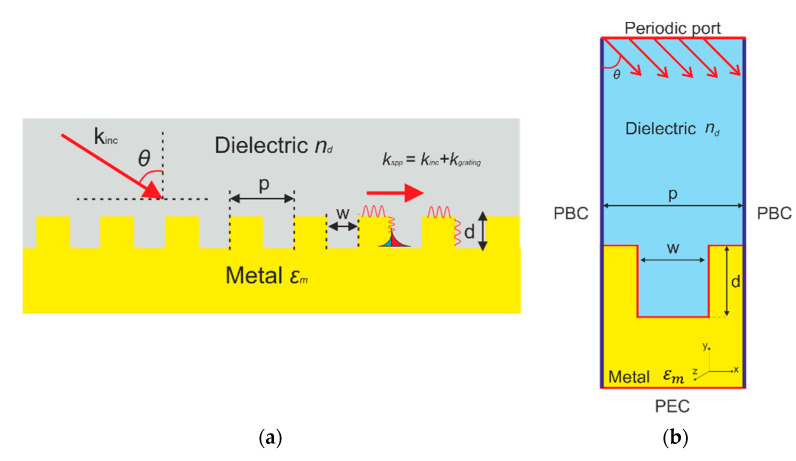
(**a**) Scheme of surface plasmon polariton resonance excitation with metal diffraction grating; (**b**) Numerical scheme to analyze SPR.

The considered structure for terahertz SPR sensing is shown in Figure 1a. It consists of the 1D linear metal diffraction grating surrounded by the analyte. The metal grating has a rectangular profile with the period p, width w, and depth d. A collimated terahertz beam with TM polarization and wavelength $\lambda_{inc}$ is incident on the sensor at the specific angle θ. After interacting with the analyte and the grating, the light is sent to the detector, which records the reflection spectra. To excite a SPR it is necessary to satisfy the momentum matching condition $k_{∥}=k_{spp}$, where $k_{∥}$ is the parallel part of the incident light wavevector $k_{inc}$ and $k_{spp}$ is the surface plasmon polariton wavevector. Upon the excitation of the SPR, a dip is observed in the reflection spectrum. Since the position of the dip depends on the properties of the analyte, the coupling frequency varies with the refractive index.

The surface plasmon polariton wavevector $k_{spp}$, propagating at the interface between the metal layer with the dielectric permittivity $\varepsilon_{m}$ and the dielectric layer with the dielectric permittivity $\varepsilon_{d}$, can be written as follows [27]:

(1) $$k_{spp}=\frac{2\pi }{\lambda_{inc}}\sqrt{\frac{\varepsilon_{m}\varepsilon_{d}}{\varepsilon_{m}+\varepsilon_{d}}}.$$

The $k_{spp}$ value is greater than the parallel part of the incident light wavevector $k_{∥}=\frac{2\pi }{\lambda_{inc}}n_{d}\sin\theta$. The diffraction grating acts like a coupler and adds the grating wavevector $m\frac{2\pi }{p}$ to $k_{∥}$. Due to this, the momentum matching condition can be rewritten as

(2) $$n_{d}\sin\theta +m\frac{\lambda_{inc}}{p}=\pm \sqrt{\frac{\varepsilon_{m}\varepsilon_{d}}{\varepsilon_{m}+\varepsilon_{d}}},$$

where m=0,±1,±2,… is the diffraction order and the sign ± on the right corresponds to the negative and positive diffraction order, respectively. In the terahertz frequency range, the dielectric permittivity of metal is high and $\varepsilon_{m}\gg \varepsilon_{d}$ [5,6], so for m=1 it follows from Equation (2) that

(3) $$p=\frac{\lambda_{inc}}{\left(1-n_{d}\sin\theta \right)}$$

### 2.2. Fabry–Perot Cavity for SPP Modes

Another resonance that can appear when an incident wave interacts with a diffraction grating is associated with the Fabry–Perot (FB) resonance. It occurs when an excited surface wave is reflected multiple times from the sidewalls of the cavity. Consistent with a free-space Fabry–Perot resonator, the resonance condition for a resonator utilizing SPPs propagating between a pair of reflective sidewalls of the cavity is satisfied when an integer number (s) of half-wavelengths fits on the length of the cavity:

(4) $$w=s\frac{\lambda_{spp}}{2}$$

### 2.3. Rectangular Waveguide Cavity Resonance Mode

In general, a deep rectangular groove is a rectangular waveguide with a cross section w×d, then the critical wavelength at which the waveguide mode can exist is

(5) $$\lambda_{ql}=\frac{2}{\sqrt{{\left(\frac{q}{w}\right)}^{2}+{\left(\frac{l}{d}\right)}^{2}}},$$

where (q,l) are the number of half-periods of the electromagnetic oscillations along the dimension w and d, respectively.

## 3. Numerical Scheme

The numerical data were obtained by the finite element method with the use of the Comsol Multiphysics software. We calculated the reflection of THz beam with frequency interrogation. The calculation scheme is shown in Figure 1b. The single groove is the unit Floquet cell with a periodic boundary condition (PBC) applied along the direction of periodicity. The excitation source is the periodic port in the boundary to inject TM-polarized terahertz radiation into the computational domain. The metal is simulated by the Drude model with the plasma frequency $\omega_{p}$ and damping frequency $\omega_{\tau }$.

The ports are used both for detection of the incident wave and for ensuring that the resulting solution leaves the model without any nonphysical reflections. For achievement of this effect, for each diffraction order m, its own port must be set. For calculation of the frequency spectra, the number of ports is determined with the condition $\left(-1-\sin\theta \right)\frac{pn_{d}}{\min\left[\lambda_{inc}\right]}\leq m\leq \left(1-\sin\theta \right)\frac{pn_{d}}{\min\left[\lambda_{inc}\right]}$. The lower part of the Floquet cell is closed with a perfect electric boundary condition (PEC). Since the penetration depth into the metal is about 50–100 nm in the terahertz range [27] and the metal grating thickness is much greater, this boundary condition has almost no effect.

## 4. Simulation Results and Discussion

### 4.1. Optimization Process

We developed sensors for gas analysis. Therefore, the refractive index of the analyte is assigned as $n_{d}=1$ for optimization process. The Drude parameters of copper was set $\omega_{p}=1.12\times 10^{16}$ rad/s and $\omega_{t}=1.38\times 10^{13}$ rad/s [28]. The incident angle and the period of the structure were chosen using Equation (3) and the condition that the number of diffraction orders will be three, including zero order. It corresponds to the maximum efficiency of SPR excitation. The period of the structure to optimize was p=175 µm and the incident angle was $\theta =15^{\circ }$, which corresponds to the resonance frequency $f_{res}=2.306$ THz ($\lambda_{res}=130$ µm).

**Figure 2:**
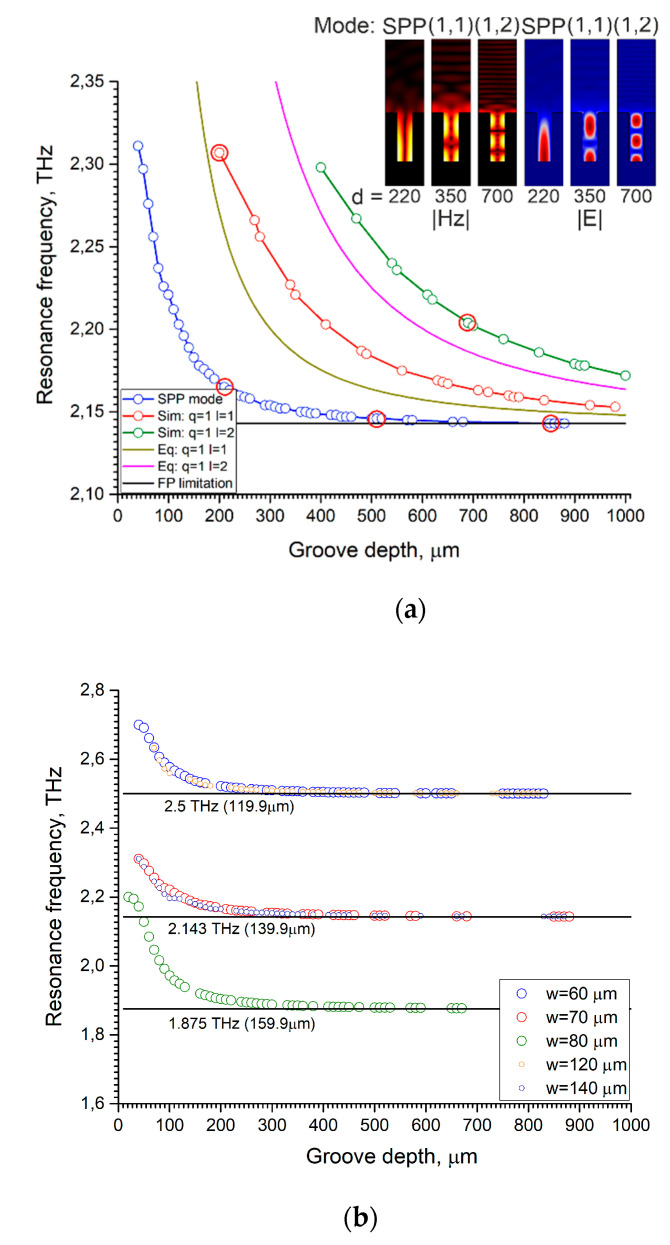
Results of calculation presented for copper diffraction gratings with rectangular profile and p=175 µm. Incident angle of TM polarized wave $\theta =15^{\circ }$. Small points on graph plots correspond to maximum dips in spectrum for given groove depth and width. (**a**) Resonance frequency vs. groove depth for different modes. Width of grooves w=70 µm. The solid lines correspond to FP resonance condition (black lines), (1, 1) mode (swamp line), and (1, 2) mode (pink line). Big red points correspond to grating parameters chosen for further analysis; (**b**) Resonance frequency vs. groove depth for SPP mode at different groove widths.

The frequency reflection spectra were calculated for different widths and depths of the grooves. Next, we caught the frequency of the dip in the reflection spectra at different parameters and plotted graphs, as shown in Figure 2a. In the reflection spectrum, several dips could be observed at once when the groove depths were more than one wavelength. Each dip corresponded to its own excited mode of grating. The small points on the graph correspond to the dip maxima in the spectrum for a given groove depth and width.

Surface plasmon polaritons at the interface of the grating surface and air appear if Equation (2) is satisfied. The excitation frequency may be lower due to the influence of higher diffraction orders and interference effects. The dip position in the frequency axis in the spectrum shifts with increases in the groove depth (Figure 2a). SPPs penetrate into the grooves along their sides and interact with the fundamental mode of the groove, which leads to the appearance of a high-intensity electric field inside the grooves.

The groove width affects the Fabry–Perot resonance condition (see Equation (4)) which entails changes in the resonant response of the grating. When the groove width is much less than the wavelength, the grating can be considered as a kind of medium with subwavelength cavities, and we did not observe deep dips in the reflection spectrum. If the groove width is $\approx \lambda_{res}/2$, resonance effects begin to appear, and modes associated with both plasmons and the Fabry–Perot effect are excited. If the groove depth is greater than the wavelength, then the grating grooves form a Fabry–Perot cavity in which plasmons interact with each other. The resonance frequency of this cavity limits the frequency of the excited plasmon in the entire system in accordance with Equation (4). The dependence of the frequency on the groove depth is repeated for multiple groove widths, as shown in Figure 2b.

In the general case, the grating groove is a rectangular resonator with a cross section w×d, and then the minimum frequency at which the mode exists can be determined from Equation (5). In calculations, we observed the excitation of resonator modes, and in some cases these resonances dominate as shown in Figure 2, and the dips in the reflection spectra were deeper for a non-SPP mode.

### 4.2. Sensor Sensitivity—Frequency Domain

**Figure 3:**
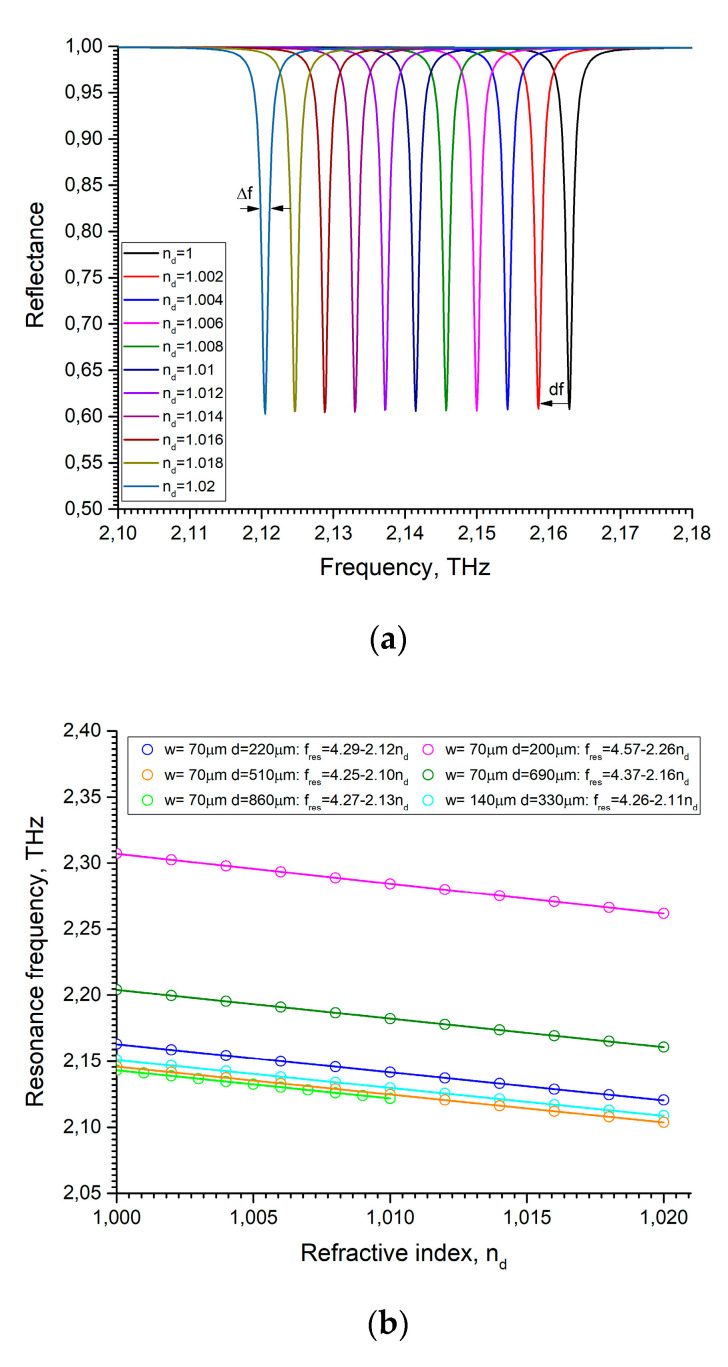
(**a**) Reflection curves of gratings filled with different refractive index analytes at p=175 μm, w=70 μm, and d=220 μm; (**b**) Resonance frequencies $n_{d}$ vs. filling refractive index for different diffraction gratings. Solid lines are linear fittings.

The main parameters that enable comparison of the sensors are the figure of merit $FOM_{f}$ and the sensitivity $S_{f}$. For the frequency spectrum, $S_{f}=df/dn$ and $FOM_{f}=S_{f}/FWHM$, where dn is the refractive index change and FWHM is the full width at half maximum of the dip in the reflection spectrum of the frequency region. To obtain the $S_{f}$ and $FOM_{f}$ parameters, we varied the refractive index, as shown in Figure 3a. Each dip corresponds to its own frequency and refractive index, and therefore it is possible to plot the dependences $f_{res}\left(n_{d}\right)$ and approximate them with $f_{res}=f_{offset}+S\cdot n_{d}$ (Figure 3b).

We can see (Figure 3b) that the resonance frequency dependences of the fundamental and higher modes remain linear with respect to the refractive index. The sensitivity of the diffraction gratings to a change in the refractive index is almost independent on the excited mode. The maximum sensitivity that we achieved in our calculations is 2.26 THz/RIU at p=175 μm, w=70 μm, and d=200 μm. The *FWHM* of the dips varies slightly with a change in the refractive index, and for an estimate it is sufficient to take the *FWHM* of the initial dip. The *FWHM* decreases with the increasing groove depth. The maximum $FOM_{f}$ reached 21250 1/RIU at p=175 μm, w=70 μm, and d=860 μm.

The detection limit of the sensor is defined as

(6) $$\delta n_{d}=\frac{\delta f_{\mathrm{res}}}{S},$$

where $\delta f_{\mathrm{res}}$ is the frequency resolution defined by the resonance *FWHM* or the spectral resolution of the radiation source. In conventional terahertz time-domain systems, $\delta f_{\mathrm{res}}\approx 5\;\mathrm{GHz}$ can be decreased to the limit of about 100 MHz, which is comparable with the best FWHM values obtained in our simulations for the deep gratings (see Table 1). The estimation according to Equation (6) gives the refractive resolution $\delta n_{d}\approx 5\times 10^{-5}\;\mathrm{RIU}$, which has the same order as in the best optical fiber refractometers in the visible and near infrared ranges [29], and as in the ultrasensitive dielectric metagratings in the subTHz domain [12]. Besides, the obtained refraction resolution is much higher than in the most sensitive THz subwavelength metal gratings using Otto prism coupling [20].

### 4.3. Sample Fabrication Method—Producibility

**Figure 4:**
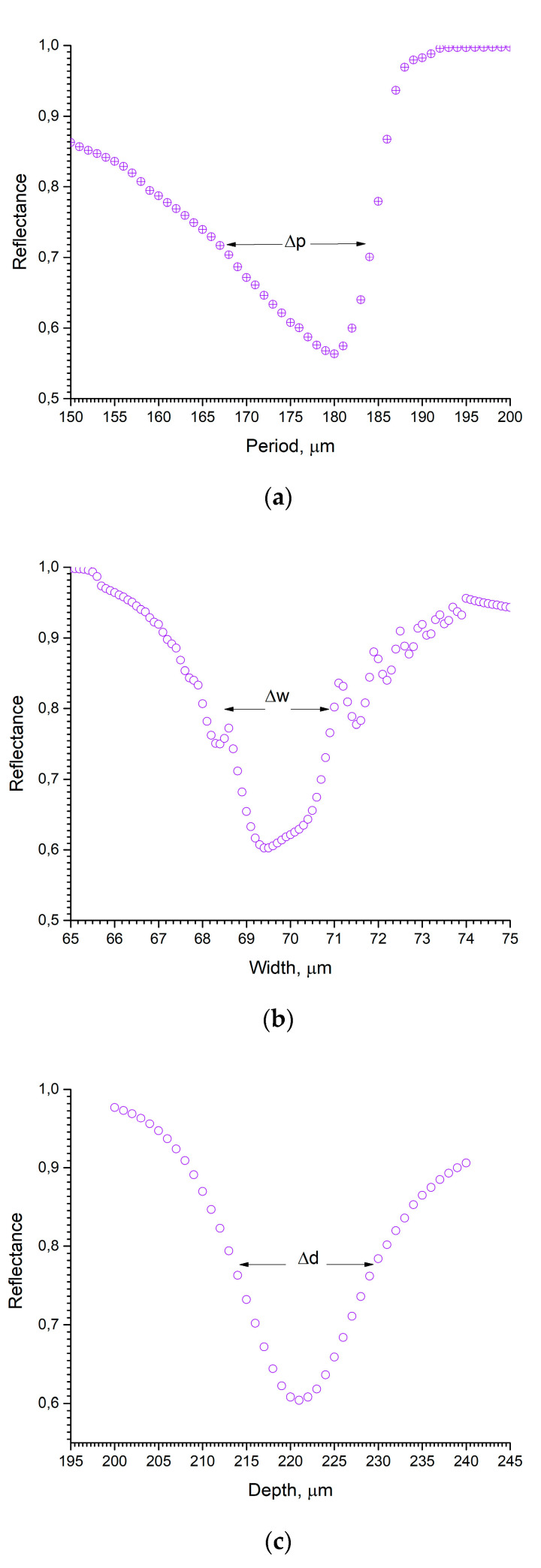
Assessment of manufacturing accuracy of grating parameters such as (**a**) period, (**b**) width, and (**c**) depth.

Diffraction gratings in the terahertz range are made by cutting grooves in a substrate using a CNC machine or a laser, compressing molding of micro-powder and 3D printing [30,31,32,33,34]. Each of these methods has its own limitations. Before using one of them, it is necessary to understand under what conditions our sensor will not be in the resonance state. To estimate it, we took one grating configuration and calculated deviation of its parameters from optimal state. As can be seen from Figure 4, the grating has the following manufacturing restrictions: p=175±13 μm, w=70±1.5 μm, d=220±5 μm. Manufacturing precision can be reached with well-developed femtosecond laser cutting machines [35], which are used in the industrial applications.

## 5. Conclusions

In this study, we consider a parameter design technique for a one-dimensional periodic rectangular metallic grating with high aspect ratio for terahertz sensing. The influence of different structure parameters on the sensing sensitivity is analyzed with the use of a numerical simulation method. The sensing sensitivity can be increased to 2.26 THz/RIU via optimization of the structure parameters when the refractive index range of the gas to measure is in the range of 1 to 1.002 with the resolution $5\times 10^{-5}\;\mathrm{RIU}$. In addition, we consider the influence of different grating modes on the sensitivity. The sensitivity of the diffraction gratings to a change in the refractive index is almost independent on the excited mode.

## Figures and Tables

**Table 1:** Parameters of diffraction gratings and their sensing possibilities.

p μm	w μm	d μm	θ ${}^{\circ }$	$f_{res},\;$ THz	R	FWHM, THz	$S_{f},\;$ THz/RIU	$FOM_{f},\;1/\mathrm{RIU}$
SPP mode								
175	70	220	15	2.163	0.621	0.00115	2.12	1843
175	70	510	15	2.146	0.617	0.0002	2.10	10,500
175	70	860	15	2.143	0.477	0.0001	2.13	21,250
(1,1)								
175	70	200	15	2.307	0.83	0.00119	2.26	1899
(1,2)								
175	70	690	15	2.204	0.768	0.001	2.16	2160
SPP mode								
175	140	330	15	2.151	0.671	0.0004	2.11	5275

## Data Availability

Not applicable.
